# Psoriasis verrugosa en un hombre con antecedentes de psoriasis en placa, reporte de caso y revisión de la literatura

**DOI:** 10.7705/biomedica.6042

**Published:** 2021-09-22

**Authors:** Javier Hernández, Ana Ruiz, Carolina Mesa, Lina Rodríguez

**Affiliations:** 1 Departamento de Dermatopatología, Clínica CES, Universidad CES, Medellín, Colombia Universidad CES Departamento de Dermatopatología Universidad CES Medellín Colombia; 2 Departamento de Dermatología, Clínica CES, Universidad CES, Medellín, Colombia Universidad CES Departamento de Dermatología Universidad CES Medellín Colombia

**Keywords:** psoriasis, carcinoma, informes de caso, psoriasis, carcinoma, case reports

## Abstract

La psoriasis verrugosa es una variante atípica y poco frecuente de la psoriasis, con pocos casos reportados en la literatura. Se caracteriza por la presencia de placas hipertróficas y verrugosas simétricas en extremidades y tronco.

Se presenta el caso de un paciente de 63 años con diagnóstico de psoriasis en placa 20 años atrás, tratado con esteroide tópico y quien 10 años antes había desarrollado una placa de aspecto verrugoso en el tercio distal de la cara posterior de la pierna izquierda. Se tomó la biopsia de la lesión por sospecha de un carcinoma escamocelular (verrugoso). El estudio histopatológico mostró cambios indicativos de psoriasis verrugosa y descartó la presencia de malignidad.

## Abstract (en)

Verrucous psoriasis is an atypical and rare variant of psoriasis with few cases reported in the literature. It is characterized by the presence of symmetric hypertrophic and verrucous plaques on the limbs and trunk.

We present the case of a 63-year-old male patient with a history of vulgar psoriasis for 20 years who was receiving treatment with topical steroids and had developed a verrucous plaque in the distal third of the posterior aspect of the leg 10 years before. We conducted a biopsy of the lesion to confirm or discard the diagnostic impression of squamous cell carcinoma (verrucous). The histopathological study showed changes compatible with verrucous psoriasis ruling out the presence of malignancy.

La psoriasis verrugosa es una variante poco frecuente de la psoriasis, con pocos casos reportados en la literatura. Clínicamente, se caracteriza por placas hipertróficas de aspecto verrugoso que suelen ser simétricas. Entre otros diagnósticos diferenciales, se incluyen el carcinoma verrugoso, las verrugas virales y el liquen plano hipertrófico.

Se presenta el caso de un paciente de 63 años con antecedentes de psoriasis en placa, quien desde hacía 10 años había desarrollado una placa verrugosa de crecimiento lento en el tercio distal de la cara posterior de la pierna izquierda.

## Reporte de caso

Se trata de un paciente masculino de 63 años con antecedentes de epilepsia, con diagnóstico de psoriasis en placas desde hacía 20 años tratada con esteroide tópico, que consultó al servicio de dermatología por aumento del número de lesiones.

**Figura 1:**
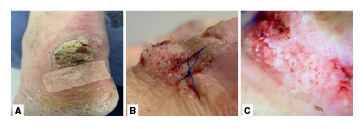
A. Placa de bordes bien definidos, regulares, de color marrón claro, hiperqueratósica y exofítica en el tercio distai de la cara posterior de la pierna izquierda. B. Placa de superficie papilomatosa posterior a la toma de la biopsia. C. En la dermatoscopia, se evidenció superficie papilomatosa blanquecina que cubría toda la extensión de la lesión.

En el examen físico, se evidenciaron placas eritematosas infiltradas con descamación plateada en codos, muslos, región interglútea y tobillos, con un índice de gravedad de la psoriasis *(Psoriasis Area Severity Index,* PASI) de 9,9 y un índice de 2 en la calidad de vida en dermatología *(Dermatology Life Quality Index,* DLQI). En el tercio distal de la cara posterior de la pierna izquierda, presentaba una lesión hiperqueratósica, exofítica y descamativa de hasta 5 cm en su eje mayor; al desprender la superficie hiperqueratósica se observó un aspecto papilomatoso sobre una base de color blanquecino (figura 1). Por la sospecha de carcinoma escamocelular (verrugoso), se tomó una biopsia de la lesión y se inició fototerapia dada la contraindicación en el paciente del tratamiento sistémico.

**Figura 2:**
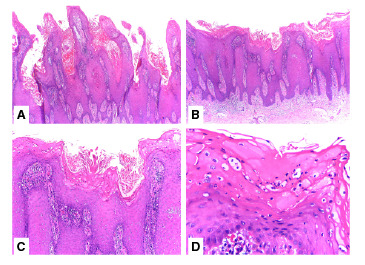
A. Papilomatosis acentuada con hiperqueratosis y acantosis epidérmica (Hematoxilina y eosina, 4X). B. Hiperqueratosis, acantosis con elongación y fusión de la red de crestas epidérmicas (Hematoxilina y eosina, 4X). C. Hiperqueratosis, agranulosis, adelgazamiento de los platos suprapapilares y vasos sanguíneos dilatados en las papilas dérmicas (Hematoxilina y eosina, 10X). D. Hiperqueratosis con microabscesos de Munro y agranulosis (Hematoxilina y eosina, 40X).

En el estudio histopatológico, se observó una lesión en piel de aspecto papilomatoso con hiperqueratosis, focos de paraqueratosis, formación de microabscesos neutrofílicos en el estrato córneo, zonas de agranulosis, acantosis con elongación de la red de crestas epidérmicas y adelgazamiento de los platos suprapapilares (figura 2). En la dermis subyacente, se detectaron vasos sanguíneos dilatados en las papilas dérmicas con infiltrado inflamatorio linfohistiocitario perivascular superficial. El estudio de inmunohistoquímica para el marcador p16 fue negativo. Se estableció el diagnóstico histológico de psoriasis de variedad verrugosa y no se encontraron signos de carcinoma escamocelular verrugoso ni cambios citopáticos que sugirieran una verruga viral.

### Consideraciones éticas

El reporte se ajustó a las disposiciones de la Resolución 008430 de 1993 del Ministerio de Salud de Colombia, según las cuales este fue un estudio sin riesgo, ya que solo se tomaron datos de la historia clínica del paciente. Asimismo, se mantuvo la confidencialidad de la información clínica y se obtuvo el consentimiento informado para la publicación del caso.

## Discusión

La psoriasis verrugosa es una variante atípica y poco frecuente de la psoriasis. Entre sus presentaciones clínicas, se encuentran la forma localizada, la eritrodérmica o la relacionada con fármacos ^1^, esta última presentada en el reporte del caso de un paciente con hepatitis C tratado con interferón alfa, que desarrolló una psoriasis verrugosa después de recibir el medicamento ^2^.

Clínicamente, esta condición se caracteriza por placas simétricas de aspecto verrugoso e hipertróficas sobre una base eritematosa ^3^ localizadas en piernas, brazos, tronco y cara dorsal de las manos ^1^. Debe sospecharse en los pacientes con antecedentes de psoriasis en placa que desarrollan de forma paulatina lesiones verrugosas, si se ha descartado un proceso infeccioso concomitante ^4^.

En el estudio histopatológico de la psoriasis verrugosa, se presenta la superposición de cambios propios de la psoriasis vulgar y de las verrugas virales ^3^^,^^5^; la piel aparece con acentuada papilomatosis, hiperqueratosis, microabscesos de neutrófilos en el estrato córneo (microabscesos de Munro), ocasionalmente agregados de neutrófilos en el estrato espinoso y acentuada acantosis con elongación de la red de crestas que, además, presentan anastomosis; en las papilas dérmicas hay vasos sanguíneos dilatados ^6^ e infiltrado inflamatorio, pero no se observan cambios coilocíticos ^3^.

La causa etiológica se desconoce, aunque se ha asociado con diabetes mellitus, disfunción pulmonar, flebitis e inmunosupresión ^3^; algunos autores la han relacionado con trastornos de la circulación linfática ^3^^,^^6^^,^^7^ y con trauma repetido en el sitio de aparición de las placas ^1^.

Entre los diagnósticos clínicos diferenciales, se debe considerar el carcinoma verrugoso, las verrugas virales, las infecciones micóticas, el liquen plano hipertrófico ^4^ y los nevus epidérmicos ^1^. La distinción entre la psoriasis verrugosa y el carcinoma verrugoso suele ser difícil, sin embargo, la ausencia de un patrón de crecimiento endofítico, sumada a la negatividad para el marcador p16, inclina la balanza a favor de la psoriasis verrugosa ^4^.

La información sobre esta variedad de psoriasis se basa en los casos reportados en la literatura. La enfermedad es de difícil manejo y no existe un consenso sobre el tratamiento que debe emplearse. Los pacientes han sido manejados con queratolíticos y esteroides tópicos de gran potencia, pero cuando se administran en monoterapia, la respuesta terapéutica ha sido deficiente ^1^^,^^3^^,^^4^. Con el uso de acitretín, etretinato, metotrexato y de la terapia biológica con etanercept, adalimumab, infliximab y ustekinumab, se ha obtenido una mejoría parcial ^1^^,^^7^. Recientemente, se describió el caso de un paciente con psoriasis verrugosa de larga data que recibió múltiples tratamientos sin éxito; finalmente, presentó mejoría con apremilast ^8^.

## Conclusión

La psoriasis verrugosa es una variante poco frecuente de la psoriasis que debe sospecharse en todos aquellos pacientes con antecedente de psoriasis que presenten placas hipertróficas de aspecto verrugoso y crecimiento lento. Se debe hacer el diagnóstico diferencial con el carcinoma verrugoso, las verrugas virales y el liquen plano hipertrófico, entre otros.
